# Acetonitrile­trichloridobis(cyclo­hexyl­diphenyl­phosphane)rhodium(III) acetonitrile disolvate

**DOI:** 10.1107/S1600536811047477

**Published:** 2011-11-19

**Authors:** Theunis J. Muller, Hendrik G. Visser, Andreas Roodt

**Affiliations:** a Department of Chemistry, University of the Free State, PO Box 339, Bloemfontein 9300, South Africa

## Abstract

In the title compound, [RhCl_3_(CH_3_CN)(C_18_H_21_P)_2_]·2CH_3_CN, the complex mol­ecule lies on a twofold rotation axis that passes through the Rh^III^ atom, one Cl atom, and the C and N atoms of the coordinated acetonitrile mol­ecule. The Rh^III^ atom is coordinated by two P atoms in *trans* positions, three Cl atoms and an acetonitrile mol­ecule in a distorted octa­hedral geometry. Intra­molecular C—H⋯Cl inter­actions are observed. The uncoordinated acetonitrile mol­ecule is disordered over two sites with occupancies of 0.588 (4) and 0.412 (4).

## Related literature

For background to the catalytic activity of rhodium–phosphane adducts, see: Brink *et al.* (2010 ▶); Marko & Heil (1974 ▶); Nagy-Magos *et al.* (1978 ▶); Oro *et al.* (1978 ▶); Roodt *et al.* (2003 ▶). For related structures, see: Archer *et al.* (1993 ▶); Aslanov *et al.* (1970 ▶); Clegg *et al.* (2002 ▶); Drew *et al.* (1970 ▶).
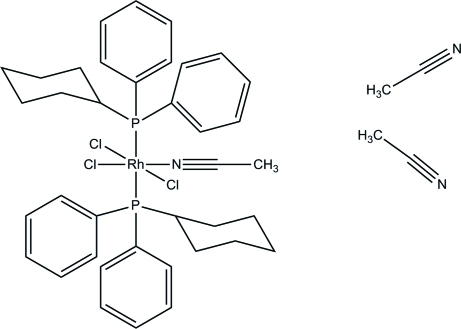


## Experimental

### 

#### Crystal data

[RhCl_3_(C_2_H_3_N)(C_18_H_21_P)_2_]·2C_2_H_3_N
*M* *_r_* = 869.06
Monoclinic, 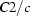

*a* = 24.995 (1) Å
*b* = 10.041 (1) Å
*c* = 16.258 (1) Å
β = 96.763 (1)°
*V* = 4052.0 (5) Å^3^
*Z* = 4
Mo *K*α radiation
μ = 0.73 mm^−1^
*T* = 100 K
0.32 × 0.25 × 0.16 mm

#### Data collection

Bruker APEXII CCD diffractometer
Absorption correction: multi-scan (*SADABS*; Bruker, 2008 ▶) *T*_min_ = 0.797, *T*_max_ = 0.889
33882 measured reflections
5038 independent reflections
4615 reflections with *I* > 2σ(*I*)
*R*_int_ = 0.027

#### Refinement

*R*[*F* ^2^ > 2σ(*F* ^2^)] = 0.022
*wR*(*F* ^2^) = 0.055
*S* = 1.04
5038 reflections
264 parameters
2 restraints
H-atom parameters constrained
Δρ_max_ = 0.37 e Å^−3^
Δρ_min_ = −0.63 e Å^−3^

### 

Data collection: *APEX2* (Bruker, 2008 ▶); cell refinement: *SAINT-Plus* (Bruker, 2008 ▶); data reduction: *SAINT-Plus*; program(s) used to solve structure: *SHELXS97* (Sheldrick, 2008 ▶); program(s) used to refine structure: *SHELXL97* (Sheldrick, 2008 ▶); molecular graphics: *DIAMOND* (Brandenburg & Putz, 2005 ▶); software used to prepare material for publication: *WinGX* (Farrugia, 1999 ▶).

## Supplementary Material

Crystal structure: contains datablock(s) global, I. DOI: 10.1107/S1600536811047477/is2783sup1.cif

Structure factors: contains datablock(s) I. DOI: 10.1107/S1600536811047477/is2783Isup2.hkl

Additional supplementary materials:  crystallographic information; 3D view; checkCIF report

## Figures and Tables

**Table 1:** Selected bond lengths (Å)

Rh1—N1	1.9978 (17)
Rh1—Cl2	2.3297 (5)
Rh1—Cl1	2.3486 (3)
Rh1—P1	2.4013 (3)

**Table 2:** Hydrogen-bond geometry (Å, °)

*D*—H⋯*A*	*D*—H	H⋯*A*	*D*⋯*A*	*D*—H⋯*A*
C10—H10⋯Cl2	0.95	2.59	3.4452 (14)	150
C20—H20*B*⋯Cl2	0.99	2.72	3.4797 (14)	134

**Fig. 1.**
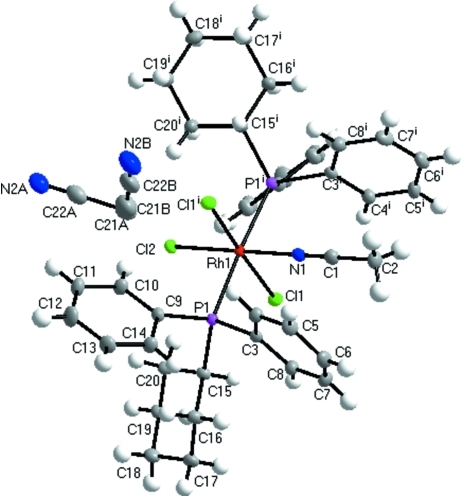
Diamond representation of the title compound, showing the numbering scheme and displacement ellipsoids (50% probability).

## supplementary crystallographic information

### Comment

Rhodium catalysts formed *in situ* from RhCl_3_xH_2_O and phosphanes have been used for the hydrogenation (Marko & Heil, 1974; Nagy-Magos *et al.*, 1978) and hydroformylation (Oro *et al.*, 1978) of olefins. The catalytic activity is determined by the electronic and steric effects of the phosphane ligand (Roodt *et al.*, 2003; Brink *et al.*, 2010).

The title compound (Fig. 1) crystallizes in the monoclinic space group *C*2/*c*. The Rh^III^ atom is situated on a twofold rotation axis, which passes atoms Cl2, N1 and C2. Two cyclohexyldiphenylphosphane ligands are positioned *trans* to each other, with the other four coordination sites occupied by three *mer*-chloroligands and one molecule of the acetonitrile solvent. In contrast to the structure reported by Clegg *et al.* (2002) the solvent molecule lies opposite the shortest Rh—Cl2 bond [2.3297 (5) Å] in the complex. Deviations from ideal octahedral geometry are minor (Table 1). The Rh—P1 bond length is 2.4013 (5) Å, while the Rh—Cl1 bond length is 2.3486 (6) Å. The P1—Rh—P1^i^ angle is 176.462 (17)° which is close to the Cl1—Rh—Cl1^i^ at 176.185 (18)° [symmetry code: (i) -*x*, *y*, -*z* + 1/2]. This complex is therefore structurally related to *trans*-ReCl_3_(PMe_2_Ph)_3_ (Aslanov *et al.*, 1970) and ReCl_3_(PPh_3_)_2_MeCN (Drew *et al.*, 1970), and other metal halide derivatives of this type (Archer *et al.*, 1993). The uncoordinated acetonitrile molecule is disordered over two positions with occupancies of 0.588 (4) and 0.412 (4). The molecular structure of the complex is stabilized by intramolecular C—H···Cl interactions (Table 2).

### Experimental

RhCl_3_.H_2_O (20 mg, 9.557×10 ^-5^ mol) was added to acetonitrile (5 ml) and heated to reflux. Cyclohexyldiphenylphosphane (2 eq, 1.911×10 ^-4^ mol, 51,2 mg) was added to the solution. The solution was refluxed for 15 min before it was cooled to room temperature. Crystals suitable for X-ray analysis was grown overnight by the slow evaporation of acetonitrile at room temperature (yield  0.0750 g, 89%)

### Refinement

H atoms were positioned geometrically (C—H = 0.93–9.97 Å) and allowed to ride on their parent atoms, with *U*_iso_(H) = 1.2*U*_eq_(phenyl C) or 1.5*U*_eq_(methyl and methylene C). The distance restraints [1.45 (1) Å] were applied for C21A—C22A and C21B—C22B.

### Crystal data

**Table:**

[RhCl_3_(C_2_H_3_N)(C_18_H_21_P)_2_]·2C_2_H_3_N	*F*(000) = 1800
*M**_r_* = 869.06	*D*_x_ = 1.425 Mg m^−^^3^
Monoclinic, *C*2/*c*	Mo *K*α radiation, λ = 0.71073 Å
*a* = 24.995 (1) Å	Cell parameters from 9837 reflections
*b* = 10.041 (1) Å	θ = 2.5–28.3°
*c* = 16.258 (1) Å	µ = 0.73 mm^−^^1^
β = 96.763 (1)°	*T* = 100 K
*V* = 4052.0 (5) Å^3^	Cuboid, red
*Z* = 4	0.32 × 0.25 × 0.16 mm

### Data collection

**Table:**

Bruker APEXII CCD diffractometer	4615 reflections with *I* > 2σ(*I*)
graphite	*R*_int_ = 0.027
φ and ω scans	θ_max_ = 28.4°, θ_min_ = 1.6°
Absorption correction: multi-scan (*SADABS*; Bruker, 2008)	*h* = −33→33
*T*_min_ = 0.797, *T*_max_ = 0.889	*k* = −13→13
33882 measured reflections	*l* = −21→19
5038 independent reflections	

### Refinement

**Table:**

Refinement on *F*^2^	Primary atom site location: structure-invariant direct methods
Least-squares matrix: full	Secondary atom site location: difference Fourier map
*R*[*F*^2^ > 2σ(*F*^2^)] = 0.022	Hydrogen site location: inferred from neighbouring sites
*wR*(*F*^2^) = 0.055	H-atom parameters constrained
*S* = 1.04	*w* = 1/[σ^2^(*F*_o_^2^) + (0.0236*P*)^2^ + 4.9122*P*] where *P* = (*F*_o_^2^ + 2*F*_c_^2^)/3
5038 reflections	(Δ/σ)_max_ = 0.001
264 parameters	Δρ_max_ = 0.37 e Å^−^^3^
2 restraints	Δρ_min_ = −0.63 e Å^−^^3^

### Special details

**Table:**

Experimental. The intensity data was collected on a Bruker X8 ApexII 4 K Kappa CCD diffractometer using an exposure time of 20 s/frame. A total of 1963 frames were collected with a frame width of 0.5° covering up to θ = 28.35° with 99.6% completeness accomplished
Geometry. All s.u.'s (except the s.u. in the dihedral angle between two l.s. planes) are estimated using the full covariance matrix. The cell s.u.'s are taken into account individually in the estimation of s.u.'s in distances, angles and torsion angles; correlations between s.u.'s in cell parameters are only used when they are defined by crystal symmetry. An approximate (isotropic) treatment of cell s.u.'s is used for estimating s.u.'s involving l.s. planes.
Refinement. Refinement of *F*^2^ against ALL reflections. The weighted *R*-factor *wR* and goodness of fit *S* are based on *F*^2^, conventional *R*-factors *R* are based on *F*, with *F* set to zero for negative *F*^2^. The threshold expression of *F*^2^ > 2σ(*F*^2^) is used only for calculating *R*-factors(gt) *etc*. and is not relevant to the choice of reflections for refinement. *R*-factors based on *F*^2^ are statistically about twice as large as those based on *F*, and *R*- factors based on ALL data will be even larger.

### Fractional atomic coordinates and isotropic or equivalent isotropic displacement parameters (Å^2^)

**Table:**

	*x*	*y*	*z*	*U*_iso_*/*U*_eq_	Occ. (<1)
Rh1	0	0.145288 (14)	0.25	0.01099 (5)	
Cl1	−0.053994 (13)	0.13750 (3)	0.35876 (2)	0.01819 (7)	
Cl2	0	0.37731 (4)	0.25	0.01635 (9)	
P1	0.079330 (13)	0.13791 (3)	0.34878 (2)	0.01236 (7)	
N1	0	−0.05368 (17)	0.25	0.0166 (3)	
C15	0.07347 (5)	0.20332 (14)	0.45392 (8)	0.0160 (3)	
H15	0.0418	0.1572	0.4734	0.019*	
C11	0.18508 (6)	0.40163 (15)	0.26099 (10)	0.0230 (3)	
H11	0.1831	0.479	0.2271	0.028*	
C2	0	−0.3095 (2)	0.25	0.0238 (4)	
H2A	0.019	−0.3421	0.2046	0.036*	0.5
H2B	−0.0372	−0.3421	0.2426	0.036*	0.5
H2C	0.0182	−0.3421	0.3029	0.036*	0.5
C5	0.14501 (6)	−0.23233 (15)	0.32610 (10)	0.0228 (3)	
H5	0.1671	−0.2744	0.29	0.027*	
C4	0.13087 (6)	−0.09925 (15)	0.31407 (9)	0.0186 (3)	
H4	0.1431	−0.0512	0.2696	0.022*	
C18	0.10192 (7)	0.36857 (16)	0.60247 (9)	0.0242 (3)	
H18A	0.1342	0.4175	0.5893	0.029*	
H18B	0.0942	0.3966	0.6582	0.029*	
C3	0.09890 (5)	−0.03580 (13)	0.36674 (8)	0.0151 (3)	
C8	0.07948 (6)	−0.10963 (15)	0.42960 (9)	0.0197 (3)	
H8	0.0562	−0.069	0.4644	0.024*	
C16	0.12161 (6)	0.17108 (15)	0.51730 (9)	0.0217 (3)	
H16A	0.1273	0.0735	0.5193	0.026*	
H16B	0.1544	0.2129	0.5	0.026*	
C10	0.13773 (6)	0.33469 (15)	0.27481 (9)	0.0205 (3)	
H10	0.1037	0.369	0.2525	0.025*	
C7	0.09408 (7)	−0.24283 (15)	0.44145 (10)	0.0250 (3)	
H7	0.0812	−0.2921	0.485	0.03*	
C19	0.05461 (6)	0.40325 (16)	0.53948 (9)	0.0220 (3)	
H19A	0.0505	0.5013	0.537	0.026*	
H19B	0.0214	0.3657	0.5579	0.026*	
C14	0.19135 (6)	0.17214 (15)	0.35757 (10)	0.0209 (3)	
H14	0.1936	0.0956	0.3922	0.025*	
C9	0.14076 (5)	0.21728 (14)	0.32148 (9)	0.0166 (3)	
C13	0.23812 (6)	0.23857 (16)	0.34300 (10)	0.0243 (3)	
H13	0.2723	0.2043	0.3647	0.029*	
C20	0.06056 (6)	0.35127 (14)	0.45333 (9)	0.0201 (3)	
H20A	0.0897	0.4009	0.4305	0.024*	
H20B	0.0267	0.3673	0.4167	0.024*	
C17	0.11302 (6)	0.22091 (16)	0.60265 (9)	0.0214 (3)	
H17A	0.0823	0.1728	0.6221	0.026*	
H17B	0.1455	0.2016	0.6418	0.026*	
C6	0.12719 (6)	−0.30370 (15)	0.39021 (10)	0.0253 (3)	
H6	0.1376	−0.394	0.3991	0.03*	
C12	0.23475 (6)	0.35541 (17)	0.29659 (11)	0.0281 (4)	
H12	0.2666	0.4036	0.2893	0.034*	
C1	0	−0.1644 (2)	0.25	0.0199 (4)	
N2A	0.22420 (12)	0.4183 (3)	0.0550 (2)	0.0384 (8)	0.588 (4)
C21A	0.2017 (4)	0.1790 (4)	0.1017 (6)	0.0345 (17)	0.588 (4)
H21A	0.1631	0.173	0.1067	0.052*	0.588 (4)
H21B	0.2223	0.1594	0.1554	0.052*	0.588 (4)
H21C	0.211	0.1144	0.0605	0.052*	0.588 (4)
C22A	0.21444 (12)	0.3123 (3)	0.07576 (19)	0.0331 (8)	0.588 (4)
N2B	0.2558 (2)	0.0458 (6)	−0.0078 (4)	0.0611 (16)	0.412 (4)
C21B	0.2042 (7)	0.1588 (10)	0.1007 (9)	0.060 (4)	0.412 (4)
H21D	0.2034	0.2552	0.0914	0.09*	0.412 (4)
H21E	0.2224	0.1399	0.1563	0.09*	0.412 (4)
H21F	0.1673	0.1243	0.096	0.09*	0.412 (4)
C22B	0.2333 (2)	0.0950 (6)	0.0394 (4)	0.0523 (17)	0.412 (4)

### Atomic displacement parameters (Å^2^)

**Table:**

	*U*^11^	*U*^22^	*U*^33^	*U*^12^	*U*^13^	*U*^23^
Rh1	0.01107 (7)	0.00977 (7)	0.01209 (7)	0	0.00121 (5)	0
Cl1	0.01540 (15)	0.02406 (18)	0.01562 (16)	−0.00089 (12)	0.00398 (12)	0.00129 (12)
Cl2	0.0172 (2)	0.0117 (2)	0.0195 (2)	0	−0.00028 (17)	0
P1	0.01149 (15)	0.01268 (16)	0.01287 (16)	−0.00007 (12)	0.00121 (12)	0.00040 (12)
N1	0.0136 (7)	0.0180 (9)	0.0173 (8)	0	−0.0018 (6)	0
C15	0.0154 (6)	0.0181 (7)	0.0141 (6)	0.0006 (5)	0.0003 (5)	−0.0021 (5)
C11	0.0214 (7)	0.0184 (7)	0.0299 (8)	−0.0017 (6)	0.0054 (6)	0.0036 (6)
C2	0.0263 (11)	0.0165 (10)	0.0285 (11)	0	0.0033 (9)	0
C5	0.0222 (7)	0.0179 (7)	0.0278 (8)	0.0047 (6)	0.0007 (6)	−0.0026 (6)
C4	0.0168 (6)	0.0190 (7)	0.0199 (7)	0.0011 (5)	0.0018 (5)	0.0012 (6)
C18	0.0279 (8)	0.0275 (8)	0.0170 (7)	−0.0023 (6)	0.0019 (6)	−0.0044 (6)
C3	0.0144 (6)	0.0146 (6)	0.0154 (6)	−0.0001 (5)	−0.0016 (5)	0.0009 (5)
C8	0.0219 (7)	0.0187 (7)	0.0185 (7)	−0.0024 (5)	0.0022 (5)	0.0010 (5)
C16	0.0238 (7)	0.0224 (7)	0.0178 (7)	0.0031 (6)	−0.0026 (6)	−0.0019 (6)
C10	0.0154 (6)	0.0212 (7)	0.0250 (7)	0.0004 (5)	0.0022 (5)	0.0044 (6)
C7	0.0322 (8)	0.0196 (7)	0.0222 (8)	−0.0048 (6)	−0.0006 (6)	0.0060 (6)
C19	0.0246 (7)	0.0207 (7)	0.0201 (7)	0.0025 (6)	0.0004 (6)	−0.0046 (6)
C14	0.0161 (6)	0.0217 (7)	0.0247 (7)	0.0002 (5)	0.0014 (5)	0.0038 (6)
C9	0.0138 (6)	0.0177 (7)	0.0185 (7)	−0.0020 (5)	0.0025 (5)	−0.0004 (5)
C13	0.0147 (6)	0.0283 (8)	0.0295 (8)	−0.0003 (6)	0.0007 (6)	0.0013 (6)
C20	0.0251 (7)	0.0184 (7)	0.0165 (7)	0.0033 (6)	0.0014 (5)	−0.0010 (5)
C17	0.0191 (7)	0.0275 (8)	0.0168 (7)	−0.0026 (6)	−0.0020 (5)	−0.0011 (6)
C6	0.0302 (8)	0.0152 (7)	0.0285 (8)	0.0014 (6)	−0.0051 (6)	0.0020 (6)
C12	0.0166 (7)	0.0297 (9)	0.0390 (9)	−0.0052 (6)	0.0073 (6)	0.0039 (7)
C1	0.0174 (9)	0.0200 (11)	0.0217 (10)	0	−0.0004 (7)	0
N2A	0.0365 (16)	0.0417 (17)	0.0367 (17)	−0.0067 (12)	0.0033 (12)	−0.0085 (13)
C21A	0.027 (3)	0.040 (3)	0.037 (4)	−0.005 (2)	0.007 (2)	−0.007 (2)
C22A	0.0247 (14)	0.0420 (19)	0.0323 (16)	−0.0032 (13)	0.0020 (11)	−0.0101 (14)
N2B	0.063 (3)	0.066 (4)	0.052 (3)	−0.027 (3)	−0.002 (3)	−0.014 (3)
C21B	0.073 (8)	0.047 (4)	0.055 (8)	−0.014 (5)	−0.021 (5)	0.009 (5)
C22B	0.056 (4)	0.049 (3)	0.046 (3)	−0.023 (3)	−0.017 (3)	0.000 (3)

### Geometric parameters (Å, °)

**Table:**

Rh1—N1	1.9978 (17)	C16—C17	1.514 (2)
Rh1—Cl2	2.3297 (5)	C16—H16A	0.99
Rh1—Cl1	2.3486 (3)	C16—H16B	0.99
Rh1—Cl1^i^	2.3486 (3)	C10—C9	1.399 (2)
Rh1—P1	2.4013 (3)	C10—H10	0.95
Rh1—P1^i^	2.4013 (3)	C7—C6	1.384 (2)
P1—C3	1.8257 (14)	C7—H7	0.95
P1—C9	1.8304 (14)	C19—C20	1.518 (2)
P1—C15	1.8529 (14)	C19—H19A	0.99
N1—C1	1.112 (3)	C19—H19B	0.99
C15—C20	1.520 (2)	C14—C13	1.390 (2)
C15—C16	1.5240 (18)	C14—C9	1.4049 (19)
C15—H15	1	C14—H14	0.95
C11—C12	1.387 (2)	C13—C12	1.392 (2)
C11—C10	1.402 (2)	C13—H13	0.95
C11—H11	0.95	C20—H20A	0.99
C2—C1	1.457 (3)	C20—H20B	0.99
C2—H2A	0.98	C17—H17A	0.99
C2—H2B	0.98	C17—H17B	0.99
C2—H2C	0.98	C6—H6	0.95
C5—C6	1.381 (2)	C12—H12	0.95
C5—C4	1.390 (2)	N2A—C22A	1.151 (5)
C5—H5	0.95	C21A—C22A	1.4501 (10)
C4—C3	1.392 (2)	C21A—H21A	0.98
C4—H4	0.95	C21A—H21B	0.98
C18—C17	1.508 (2)	C21A—H21C	0.98
C18—C19	1.511 (2)	N2B—C22B	1.119 (9)
C18—H18A	0.99	C21B—C22B	1.4498 (10)
C18—H18B	0.99	C21B—H21D	0.98
C3—C8	1.395 (2)	C21B—H21E	0.98
C8—C7	1.394 (2)	C21B—H21F	0.98
C8—H8	0.95		
			
N1—Rh1—Cl2	180	C17—C16—C15	111.33 (12)
N1—Rh1—Cl1	88.093 (9)	C17—C16—H16A	109.4
Cl2—Rh1—Cl1	91.907 (9)	C15—C16—H16A	109.4
N1—Rh1—Cl1^i^	88.093 (9)	C17—C16—H16B	109.4
Cl2—Rh1—Cl1^i^	91.907 (9)	C15—C16—H16B	109.4
Cl1—Rh1—Cl1^i^	176.185 (18)	H16A—C16—H16B	108
N1—Rh1—P1	88.231 (9)	C9—C10—C11	119.86 (13)
Cl2—Rh1—P1	91.769 (9)	C9—C10—H10	120.1
Cl1—Rh1—P1	89.879 (12)	C11—C10—H10	120.1
Cl1^i^—Rh1—P1	90.003 (12)	C6—C7—C8	120.40 (15)
N1—Rh1—P1^i^	88.231 (9)	C6—C7—H7	119.8
Cl2—Rh1—P1^i^	91.769 (9)	C8—C7—H7	119.8
Cl1—Rh1—P1^i^	90.003 (12)	C18—C19—C20	113.09 (13)
Cl1^i^—Rh1—P1^i^	89.879 (12)	C18—C19—H19A	109
P1—Rh1—P1^i^	176.463 (17)	C20—C19—H19A	109
C3—P1—C9	103.78 (6)	C18—C19—H19B	109
C3—P1—C15	103.88 (6)	C20—C19—H19B	109
C9—P1—C15	103.24 (6)	H19A—C19—H19B	107.8
C3—P1—Rh1	108.72 (4)	C13—C14—C9	120.50 (14)
C9—P1—Rh1	118.33 (5)	C13—C14—H14	119.8
C15—P1—Rh1	117.21 (4)	C9—C14—H14	119.8
C1—N1—Rh1	180	C10—C9—C14	119.15 (13)
C20—C15—C16	111.22 (12)	C10—C9—P1	120.32 (10)
C20—C15—P1	112.36 (10)	C14—C9—P1	119.84 (11)
C16—C15—P1	113.99 (10)	C14—C13—C12	119.89 (14)
C20—C15—H15	106.2	C14—C13—H13	120.1
C16—C15—H15	106.2	C12—C13—H13	120.1
P1—C15—H15	106.2	C19—C20—C15	111.96 (12)
C12—C11—C10	120.26 (14)	C19—C20—H20A	109.2
C12—C11—H11	119.9	C15—C20—H20A	109.2
C10—C11—H11	119.9	C19—C20—H20B	109.2
C1—C2—H2A	109.5	C15—C20—H20B	109.2
C1—C2—H2B	109.5	H20A—C20—H20B	107.9
H2A—C2—H2B	109.5	C18—C17—C16	111.65 (13)
C1—C2—H2C	109.5	C18—C17—H17A	109.3
H2A—C2—H2C	109.5	C16—C17—H17A	109.3
H2B—C2—H2C	109.5	C18—C17—H17B	109.3
C6—C5—C4	120.38 (15)	C16—C17—H17B	109.3
C6—C5—H5	119.8	H17A—C17—H17B	108
C4—C5—H5	119.8	C5—C6—C7	119.61 (14)
C5—C4—C3	120.53 (14)	C5—C6—H6	120.2
C5—C4—H4	119.7	C7—C6—H6	120.2
C3—C4—H4	119.7	C11—C12—C13	120.15 (14)
C17—C18—C19	110.91 (12)	C11—C12—H12	119.9
C17—C18—H18A	109.5	C13—C12—H12	119.9
C19—C18—H18A	109.5	N1—C1—C2	180
C17—C18—H18B	109.5	N2A—C22A—C21A	179.5 (5)
C19—C18—H18B	109.5	C22B—C21B—H21D	109.5
H18A—C18—H18B	108	C22B—C21B—H21E	109.5
C4—C3—C8	118.80 (13)	H21D—C21B—H21E	109.5
C4—C3—P1	120.07 (11)	C22B—C21B—H21F	109.5
C8—C3—P1	121.00 (11)	H21D—C21B—H21F	109.5
C7—C8—C3	120.21 (14)	H21E—C21B—H21F	109.5
C7—C8—H8	119.9	N2B—C22B—C21B	179.9 (11)
C3—C8—H8	119.9		

Symmetry codes: (i) −*x*, *y*, −*z*+1/2.

### Hydrogen-bond geometry (Å, °)

**Table:**

*D*—H···*A*	*D*—H	H···*A*	*D*···*A*	*D*—H···*A*
C10—H10···Cl2	0.95	2.59	3.4452 (14)	150.
C20—H20B···Cl2	0.99	2.72	3.4797 (14)	134.
